# Richmond Academy of Medicine and Surgery

**Published:** 1890-09

**Authors:** Wm. W. Parker, J. W. Henson

**Affiliations:** President, in the Chair; Reporter


### RICHMOND ACADEMY OF MEDICINE AND SURGERY. STATED MEETING JULY 8TH.

DR. WM. W. PARKER, PRESIDENT, IN THE CHAIR.
J. W. HENSON, M. D., Reporter.

### SPEECH AND LOCOMOTION ABSENT IN A CHILD OF THREE AND A-HALF YEARS OF AGE.

Dr. J. N. Upshur reported the case of a child unable to walk or talk at the age of three and a-half years, although apparently perfectly developed physically, and to a casual observer as bright mentally as any child—in reality, however, being several months or a year behind the average. The expression of its face was a little more childish than the age demanded.

There was, he said, a remarkable suppleness about the hip joints, the child being able to abduct the lower limbs until at right angles with the trunk, or flex them until flat upon the abdomen.

It possessed a good appetite, was perfectly well nourished, though constipated, and had resisted well two or three severe spells of illness. It had a remarkable aptitude for the appreciation of musical sounds. The child's teeth exhibited great irregularity in their manner of eruption, appearing here and there at haphazard around the dental arch.

The doctor knew of no cause for the state of affairs except that the mother, when pregnant with this child, was subjected to considerable mental and physical worry on account of the illness of an older one. He would like to know the chances of its attaining speech and locomotion.

Was the condition the result of lack of nervous power, and would benefit accrue from the use of electricity and massage?

Dr. J. Michaux—Had there been any convulsions?

Dr. Upshur—None.

Dr. C. L. Cudlipp—Were all the pelvic bones normal?

Dr. Upshur—Yes.

Dr. Michaux thought the case one of arrest of development from lack of brain or nervous organization, and that there was little chance for mental development under such conditions.

The President, Dr. Parker, thought a child of three years would learn to talk.

Dr. Geo. Ben. Johnston believed the case, from the history, one of mild rickets, and was sure that by an active tonic treatment in which the hypophosphites were involved, massage (particularly), electricity and strict attention to hygienic surroundings, much good could be done for the child's bones· He thought it would walk, and did not believe the inability to speak necessarily serious.

VERATRUM VIRIDE IN PUERPERAL CONVULSIONS.

Dr. Parker reported having used, in a case of puerperal convulsions occurring two or three weeks before the expected time of labor (besides the usual plan of venesection and chloroform), tincture of veratrum viride, administering 14 gtts. early, and afterwards 5 gtts. every two hours.

Dr. Hugh M. Taylor, in consultation recommended enemata of bromide of potassium and hydrate of chloral, in large doses.

The patient was successfully relieved, but labor commenced two or three days afterwards, and under chloroform she gave birth to a live child of eight months gestation, large but feeble.

The doctor (Parker) had great faith in veratrum viride for the relief of puerperal convulsions. Dr. Albert Sneed had recommended it in ten-drop doses every two hours.

CHOLERA MORBUS RAPIDLY FATAL.

A Mr. V. summoned medical aid about 2 a. m. on Wednesday. By 3 p. m. on Thursday he was dead.

Before death the vomiting and purging became excessive, and a convulsive movement of the lower extremities manifested itself.

The victim had been robust and perfectly healthy all of his life except an anal fistula some years ago. Dr. Parker had been the family physician, but being out of town, another doctor was called, who reported the case to him.

Dr. Parker thought the action of the vagus nerve had been inhibited by the intense heat, the man's work keeping him much in the sun.

CHLOROFORM VS. OPIUM IN INTESTINAL INFLAMMATIONS.

A short time after V's. death, continued Dr. Parker, his son was stricken down.

After the first day or two of illness he complained of very little pain.

The doctor, accepting the case only the day before death occurred, found him quiet, pulse 120 and temperature 101°, but though there was no pain except upon deep pressure, it was then severe, and the abdomen was retracted—two bad features. Late the next day the boy was in collapse, death soon following.

A post mortem examination revealed the ascending colon pushed obliquely across the abdomen by the greatly distended and inflamed small intestines, which here and there showed adhesions and exudations (some as large as a fifty cent piece), about to undergo organization.

In fact, a severe general peritonitis had existed, a pint of pus being in the cavity of the peritonaeum.

The doctor believed the lack of pain due to the amount of morphine given by the physician first in charge. He objected to such large doses of the drug, and mentioned in connection a fatal case of intestinal inflammation to which he had been called at Old Point. The physicians called in before him had probably administered large doses of morphine. He found the man in collapse, perfectly quiet and indifferent.

No amount of stimulation or other means used produced any reaction.

He believed large doses of opium would not only prevent reaction, but increase congestion. He thought the pain of these cases very largely due to spasm of the muscular layer of the bowel, and therefore would just as readily and much more safely be relieved by chloroform (by inhalation and internally), together with stimulants.

Dr. Johnston asked if there was any debris of food in the colon, particularly about the cæcum, in the post mortem case mentioned.

Dr. Parker—None.

IRRITATION FROM CALOMEL AND CASTOR OIL.

Dr. Upshur believed there was something back of the opium in Dr. Parker's case. He thought the purgative action from large doses of calomel (such as 15 grs.) and the castor oil following it would add to the irritation and congestion.

The kind of congestion referred to by Dr. Parker would be aggravated by opium, but he considered the drug beneficial in passive congestions, such as occurred in the latter stages of typhoid fever.

STILL BORN CHILD EXPECTED AFTER CONVULSIONS.

Dr. Upshur was interested in Dr. Parker's case of puerperal convulsions because the child was born alive.

He always expected a dead child after convulsions.

EXAMINATION FOR URAEMIC SYMPTOMS DURING PREGNANCY IMPERATIVE—PILOCARPINE IN URAEMIA.

The doctor believed it the *imperative* duty of every physician to make periodical examinations of the urine of pregnant women in his charge, and to enquire into the amount of water passed per day, and the condition of head and vision.

There might be double vision, intense headache and scanty urine, without albumen, and yet convulsions. He remembered a patient of his who complained of severe headache two weeks before confinement, no albumen being present and no impairment of vision.

Just after completion of labor she was threatened with convulsions. The prompt and continued use of chloroform, however, warded off the attack.

The skin was hot and dry.

Bromide of potassium and pilocarpine were administered in repeated doses until a profuse perspiration was induced, with relief of head symptoms. Examination of urine now showed 33 per cent of albumen.

The patient made a complete recovery.

He mentioned another case in which he had the same experience with pilocarpine.

He knew the objection to it—that it was depressing—but why object to it and recommend veratrum viride.

For the immediate relief of convulsions he used morphine and atropia hypodermically, besides the lancet and chloroform.

Dr. Landon B. Edwards thought Dr. Upshur had given the true cause why some physicians had so many cases of puerperal convulsions.

The maxim of Dr. Owen, of Lynchburg, was watch the woman as you would the training of a child.

Though convulsions did not always follow the symptons, yet those symptoms should be treated as warnings.

As a prominent symptom he mentioned the morbid appetite in the latter stages of pregnancy. First quiet the alarm of the patient, then direct attention to the kidneys. He, too, highly recommended pilocarpine if the patient was strong enough to cough up or call attention to the accumulation that would occur in the bronchial tubes.

If a convulsion occurred she might drown them.

ERRATIC PAIN IN LABOR.

Dr. Johnston had been called fifteen or twenty days before her expected delivery, to a woman, the mother of four children (good labor each time), who complained of a severe pain, paroxysmal in character, occurring on the right side of the neck, and extending down upon her chest to the margin of the axilla.

The doctor, suspecting the approach of labor, asked an examination, but was refused.

Early the next morning he was called again, and found a child born.

The pains had increased in length and intensity, the intervals growing shorter, until there was suddenly a gush of waters, the birth of the child immediately following.

The woman hadn't a single uterine or abdominal pain, and did not in the least suspect the real condition of affairs.

Dr. Parker—Was the woman intelligent?

Dr. Johnston—Very.

Dr. Parker—Was she of neuralgic tendency?

Dr. Johnston—No.

SCIRRHUS OF THE RECTUM IN A CHILD OF THIRTEEN YEARS.

Dr. Michaux had been treating a child of 13 years of age for ulcerated rectum, for some time, with no benefit. He decided upon an examination of the parts, which he made, with the patient under chloroform. About two inches above the anus he found a band of two and a half inches in width, nearly closing the calibre of the bowel.

It was hard to the touch, but tore upon pressing the finger through it.

There was some inguinal enlargement.

Every motion of the bowels caused violent pain, and this examination induced so much as to necessitate the use of opiates.

The general appearance of the boy suggested malignancy, and the doctor believed it such, though he had never seen or known of a case in so young a subject.

Dr. Parker—Any sanious discharge?

Dr. Michaux—Some mucus and pus, probably from inflammation around the growth.

Dr. Upshur—Was there a history of chronic constipation?

Dr. Michaux—No.

Dr. John R. Wheat—Any history of syphilis?

Dr. Michaux—No.

Dr. Upshur—Any history of dysentery?

Dr. Michaux—Thought not, except that depending upon the present trouble.

Dr. Upshur—What was the character of the pain?

Dr. Michaux—Very acute.

Dr. Upshur—Much pain at night?

Dr. Michaux—No.

Dr. Upshur, refusing to believe in malignancy at that age, thought Dr. Michaux would find that some previous proctitis had produced the band of lymph present, or that there was some history of syphilis back of the trouble. He had seen such a case in a woman of decided syphilitic history, there being acute pain upon defecation. He performed repeated cuttings and dilatations. Her health ultimately gave out, death following.

He would suggest alteratives, such as iodide, iron, etc., and nutritious but fluid diet. The rectum might be washed out with warm water and boracic acid. The pain could be relieved by suppositories medicated with cocaine or enemata of glycerine and cocaine.

Dr. Wheat thought Dr. Michaux had better look after a probable syphilitic history. He related two cases of his own.

He had found that a constitutional treatment, involving potassium, iodide particularly, gave decided relief.

Though the trouble returned, this treatment relieved each time.

He had no faith in operative measures in such cases. Had tested that plan.

Dr. Michaux had neglected to say the child's grandfather died of cancer.

He would, however, take advantage of the encouraging suggestions.

He would obtain some of the growth for microscopic examination.

[Since the above meeting Dr. Upshur found, upon stripping the little girl of three and a half years, whose condition he reported, that there was a uniform atrophy of the muscular system. He has given her the benefit of massage and electricity for ten days. Improvement has manifested itself by the more ruddy appearance generally, and the toning up of the muscles.]

---

ECZEMA.—Dr. Mackintosh gives the following as an ointment, which in most cases pretty nearly approaches the character of a specific: ℞. Bismuthi Subnitratis, 4 drachms; Zinc Oxidi, 1 drachm; Acid Carbolic Liquidi, 1-2 drachm; Vaselini Albi, 2 ounces; Ft. ung. Or, ℞. Bismuthi Subnitratis, 3 drachms; Zinc Oxidi, 1-2 drachm; Glycerini (Price's), 1 1-2 drachms; Acidi Carbolici Liquidi, 20 minims; Vaselini Albi, 6 drachms; Ft. ung.

The latter ointment mixes into a beautiful enamel-like cream, which is cooling, and acts as a balm to the irritable skin. When constant tingling and irritation disturb the patient's rest at night the following lotion is said to be valuable: ℞. Bismuthi Subnitratis, 1 drachm; Glycerini (Price's), 4 drachms; Acidi Carbolici Liquidi, 12 minims; Aquæ Rosæ, ad., 1 ounce. M. Sig.—Shake up and apply with a camel's hair pencil.—*Omaha Clinic.*

